# *In situ* growth of novel carbon nanobuds and nanoballs on graphene nanosheets by the electrochemical method

**DOI:** 10.1039/d2ra01695h

**Published:** 2022-06-17

**Authors:** Qi Qin, Jing Chen, Changze Wu, Yixue Wang, Yunan Li, Meng Song

**Affiliations:** School of Materials and Chemical Engineering, Zhongyuan University of Technology 41 Zhongyuan Road Zhengzhou 450007 P. R. China qq@zut.edu.cn +86 19937673288

## Abstract

Novel carbon nanostructures, carbon nanobuds and nanoballs *in situ* grown on graphene, have been synthesized by the electrochemical method in this study. Pristine graphene (GR) sheets were potentiostatic treated with sulfuric acid and were oxidized at 1.4–2.0 V constant potentials to obtain numerous nanobuds and peeled nanoballs. Scanning electron microscopy was used to determine the morphology of electrochemically treated GR nanosheets. Fourier transform infrared, X-ray diffraction analysis, and Raman spectroscopy were used to characterize the structure of samples. The above results indicate that amounts of nanobuds were *in situ* grown on the surface of GR sheets at a constant potential of 1.4 V was added to the GR electrode. With the constant potential increasing, the nanobuds grew into the nanoballs, exfoliating from the surface of graphene sheets, whereas the peroxidation of graphene sheets occurred at a higher potential of 2.0 V, leading to the formation of a large amount of graphene oxide fragments. Therefore, the optimal processing parameter of the formation of carbon nanoballs was under the constant potential of 1.8 V for 500 s.

## Abstract 2

Novel carbon nanostructures, carbon nanobuds and nanoballs *in situ* grown on graphene, have been synthesized by the electrochemical method in this study.

## Graphical abstract

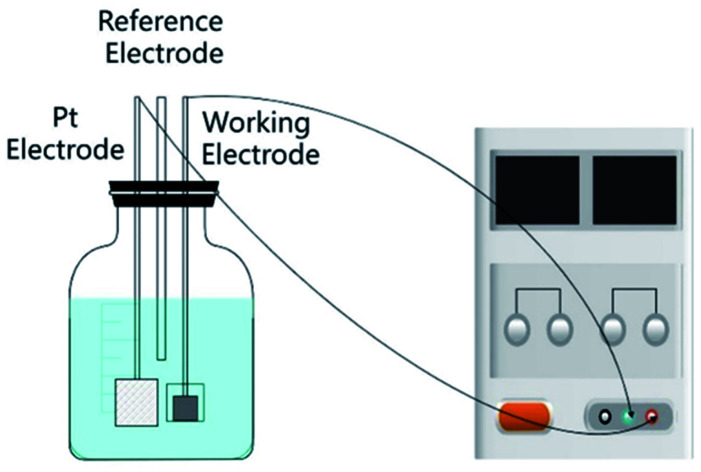

## 1. Introduction

Carbon is one of the earliest elements that humans have come into contact with and one of the earliest elements that humans have utilized. There are many isomers of carbon in nature, such as diamond, graphite, fullerenes, carbon nanotubes, graphene, *etc.*, all of which have left an indelible mark on the history of mankind. Diamond and graphite have been studied by humans as the earliest isomers of carbon. Since the discovery of fullerenes by Kroto *et al.* in 1985, new structures of fullerenes have been continuously predicted or discovered beyond the individual clusters themselves.^1–3^ The emergence of fullerenes opened the door to a new set of nanoscale carbon isomers, and fullerenes are considered to be the third isomer of carbon after the discovery of diamond and graphite.^4,5^ They consist of nearly 60 carbon atoms, denoted by C_60_. In 1991, Iijima discovered carbon nanotubes which played an important role in the field of polymer composites.^6,7^ In recent years, as the research on carbon nanotubes and nanomaterials has intensified, their promising applications have continued to show.^8^ In 2004, two scientists from the University of Manchester, UK, Geim and Novoselov, discovered that they could obtain increasingly thin graphite sheets in a very simple way. Finally, they obtained a sheet consisting of only one layer of carbon atoms, which is graphene.^9–11^ Graphene is a new material in which carbon atoms connected by sp^2^ hybridization are tightly packed into a single layer of two-dimensional honeycomb lattice structure. With excellent optical, electrical, and mechanical properties, graphene holds significant promise for applications in materials science, micro and nano processing, energy, biomedicine, and drug delivery and is considered a revolutionary material of the future.^12,13^ However, for each of these materials there are their drawbacks and challenges. For example, when functionalizing carbon nanotubes or fullerenes, it usually destroys its kind of perfect structure and thus its excellent properties. With the development of carbon isomers and the endless attempts of scientists to combine two or more different carbon nanostructures with each other, a number of new carbon nanohybrids have been created. Among them, carbon nanobuds are the brightest product. They are generated by attaching a fullerene to a carbon nanotube. Both experiments and theories show that carbon nanobuds are more easily functionalized. Theoretical simulations also show that the electrical conductivity of the nanobud depends on the details of its geometry.^14^ Despite their relative novelty in the scientific community, carbon nanobuds have shown their potential as a useful nanomaterial. Field emission measurements suggest that they may be advantageous compared to carbon nanotubes or fullerenes alone. With that said, fullerenes, carbon nanotubes and graphene, the three most famous sp^2^ nanocarbons, have generated a great deal of interest in the scientific and technological community. Given the extensive research on these amazing nanocarbons, these nanocarbons show great promise in a wide variety of applications ranging from high-performance composites to electronic and energy storage devices to biomaterials.^15–18^ Researchers have never stopped moving forward on the road to a new era of nanocarbon. Carbon nanobuds (CNB), a novel carbon nanostructure, have been synthesized recently *via* covalently bonding C_60_ buckyballs to the sidewall of a single-walled carbon nanotube (SWCNT) through cycloaddition reaction by Nasibulin^19^*et al.*

As mentioned above, fullerenes, carbon nanotubes, graphene and graphite have a similar structure and electronic properties, and graphene composed of an sp^2^ hybridized carbon bond is a basic building block of CNT, fullerenes, and graphite. Hence, we consider that there would be a structural transformation from graphene to other allotropes of carbon. Some mechanism or theory with respect to this translation of the different allotropes of carbon has been approved. Uberuaga^20^*et al.* studied the fullerene and graphene formation from carbon nanotube fragments by longtime annealing using the accelerated molecular dynamics methods. These results suggest that fragments from nanotubes exhibit surprising behavior, unfolding to form thermodynamically unfavorable graphene fragments. Manna^21^ has reported computational studies on non-covalent interactions of carbon and boron fullerenes with graphene. Chuvilin^22^ has reported the direct transformation of graphene to fullerene; scanning electron microscopy directly visualized a process of fullerene formation from a graphene sheet. Georglk *et al.*^23^ successfully prepared carbon nanobuds using pristine graphene nanosheets (pG) and C_60_ as reactants. The prepared graphene nanobuds exhibited significantly improved dispersion and abundant aromatic properties, which also provided higher electrical conductivity.

Although graphene and fullerenes are interconvertible in principle, a simply viable route to translation from a flat graphene sheet to these carbon cages remains a mystery. In this article, direct evidence for the formation of a novel carbon hollow sphere from graphene nanosheets by the electrochemical method will be reported. Carbon nanobuds and fullerenes can be efficiently generated from graphene under the potentiostatic electric field, and this process can be observed with a scanning electron microscope.

## 2. Experimental

### 2.1 Materials

Graphene nanosheets (C > 99.5 wt%) were bought from Nanjing Xianfeng Nanomaterials Technology Co., Ltd. ITO-PET conducting films (Shenzhen Huanan Xiangcheng Science and Technology Co., Ltd. 10–30 Ω □^−1^) were used for GR deposition. Analytical grade reagents, H_2_SO_4_ (98 wt%) and ethanol (Sinopharm Chemical Reagent Co., Ltd.) were used without any pretreatment. All solutions were prepared from de-ionized water.

### 2.2 Preparation of a GR flexible electrode

Firstly, the pretreatment of ITO-PET substrates retained a 1 × 1 cm^2^ effective area of this electrode with other areas insulated by an adhesive tape coating. Then ITO-PET films were rinsed with de-ionized water and ethanol before graphene paint. Secondly, certain weight graphene was dispersed in 10 ml de-ionized water ultrasonically for 40 min until the solution was mixed well-distributed. Thirdly, graphene solution was dropped on the ITO-PET plate by a doctor scraping technique to form a black thin film and dried in a vacuum at 80 °C for 30 min. Then the third step was repeated 5 times until the graphene film spread on the PET substrate reached a certain thickness. Finally, a GR flexible electrode coated on a conducting PET film has been prepared.

### 2.3 Electrochemical process of the GR electrode

**Fig. 1:**
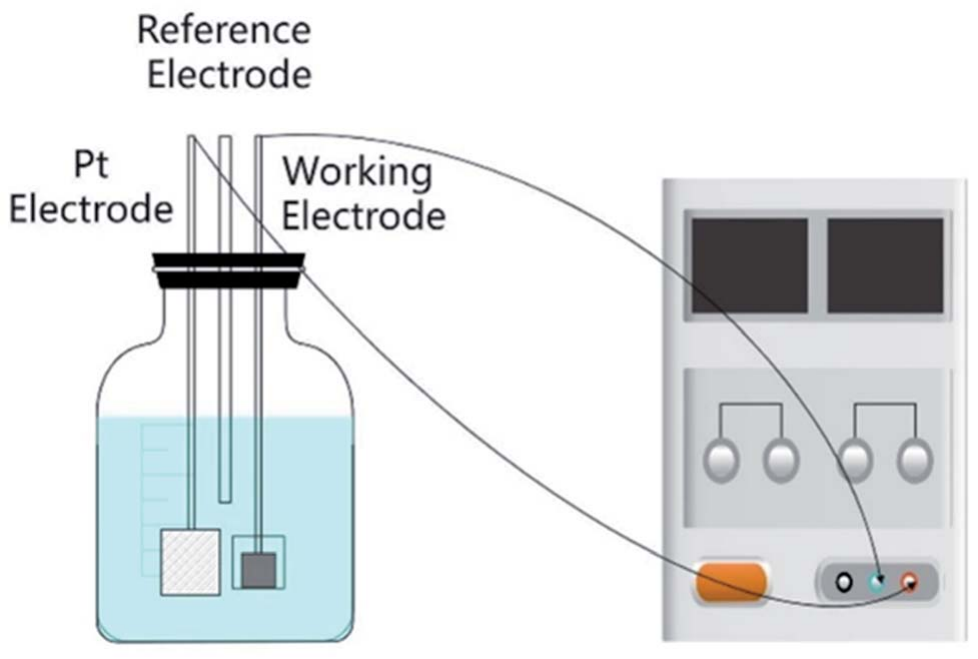
Schematic diagram of the electrochemical device.

The electrochemical process of the GR electrode was performed by the constant potential technique from an electrolyte consisting of 0.5 mol L^−1^ H_2_SO_4_ in a three-electrode cell. The three-electrode cell was constructed with the above graphene electrode as the working electrode, a Pt sheet as the counter electrode and a saturated calomel electrode (SCE) as the reference electrode. The potentiostatic treatment was carried out from 1.4 V to 2.0 V for 500 s using a computer-controlled electrochemical analyzer (CHI660C, Shanghai CH instruments, China). Finally, these resultant GR-PET electrodes were cleaned with deionized water several times and dried at 60 °C in a vacuum for 24 h (Fig. 1).

### 2.4 Measurement and characterization

The micromorphologies of GR electrodes were observed by field-emission scanning electron microscopy (FE-SEM, JEOL JSM-7001F, Japan) equipped with energy disperse spectroscopy (EDS, Oxford Instruments). Fourier transform infrared (FTIR) spectra were recorded in the range of 500–4000 cm^−1^ with an FTIR spectrometer (Nicolet iS10, USA). The phase composition was identified by X-ray diffraction (XRD, XD-3, Beijing Purkinje General Instrument Co., Ltd, China) at a scanning speed of 10° min^−1^ and a scanning range of 5°∼70°.

## 3. Results and discussion

### 3.1 Morphological properties

**Fig. 2:**
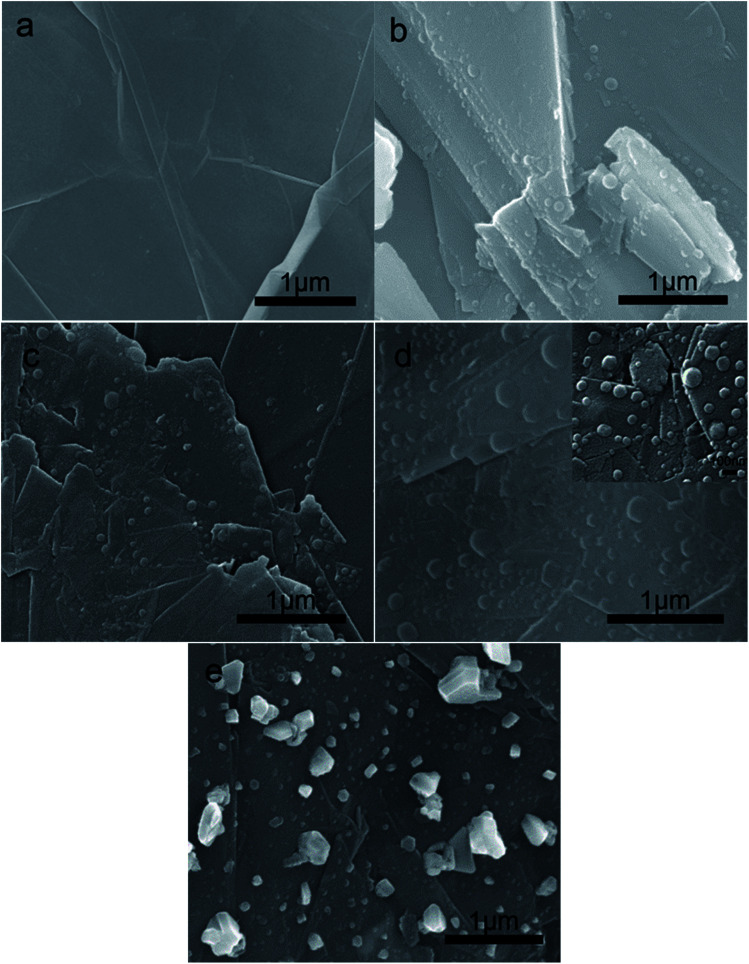
SEM photos of (a) GR sheets and GR potentiostatic treated at (b)1.4 V, (c) 1.6 V, (d)1.8 V, and (e)2.0 V.

The morphologies of GR films potentiostatic treated at different potentials are shown in Fig. 2. From Fig. 2(a), we can see the two-dimensional sheets of graphene. The surface of graphene sheets was relatively smooth and flat. When a constant potential was added to the GR electrode, it could be found that many small nanobuds grew on the surface of the GR sheet from 1.4 V to 2.0 V. With the constant potential increasing, the nanobuds grew bigger and bigger, and then formed the carbon nanoballs, exfoliating from the surface of graphene sheets.

A large number of nanobuds and several nanoballs grown on the local area of GR sheets at 1.4 V are shown in Fig. 2(b). The size of most nanobuds was less than 10 nm, and a little nanoball grew bigger to 20–30 nm. As the constant potential increased to 1.6 V, there were more nanobuds and nanoballs formed on the larger area of GR sheets. The amount and size of nanoballs at 1.6 V were much larger than those at 1.4 V. Then as the constant potential increased to 1.8 V, carbon nanoballs grew bigger, and the diameter was more than 100 nm. These nanospheres were almost covered with GR nanosheets, some of them were embedded in the nanosheets, and some of them were peeling off the nanosheets.

Quantum chemical modeling explains four critical steps in a top-down mechanism of nanosphere formation: (i) loss of carbon atoms at the edge of graphene under the electric field, leading to (ii) the formation of pentagons, which (iii) causes the curving of graphene into a bowl-shaped structure, like graphene ‘buds’ and which (iv) subsequently zips up its open edges to form a closed sphere structure. This is the most widely accepted mechanism of the two-dimensional graphene layer structure dissociating into very small clusters of carbon nanoballs such as C_60_. In this article, scanning electron microscopy directly visualizes, in real-time, a process of carbon nanoball formation from a graphene sheet.

As the constant potential increased to 2.0 V, instead of nanospheres, irregular bulks were formed under higher potential as shown in Fig. 2(e). The size of graphene bulks ranges from tens to hundreds of nanometers. This formation mechanism of irregular nano bulks was deduced as follows: higher electric field energy (above 1.8 V) can trigger the graphene fragmentations stripped from the nanosheets to aggregate into small nanoclusters, which subsequently coalesce to form large and irregular bulks through a series of intermediates. The fragmentation of the embedded molecules treated at elevated potential then produces carbon clusters that undergo diffusion and aggregation to form graphene bulks.

Therefore, the shape and size of the carbon clusters embedded in graphene nanosheets can be tailored by optimizing the constant potential. In conclusion, the formation of carbon nanoballs shows that the best constant potential was 1.8 V for 500 s, confirming that nanoballs were very stable at room temperature.

### 3.2 Potentiostatic treatment

**Fig. 3:**
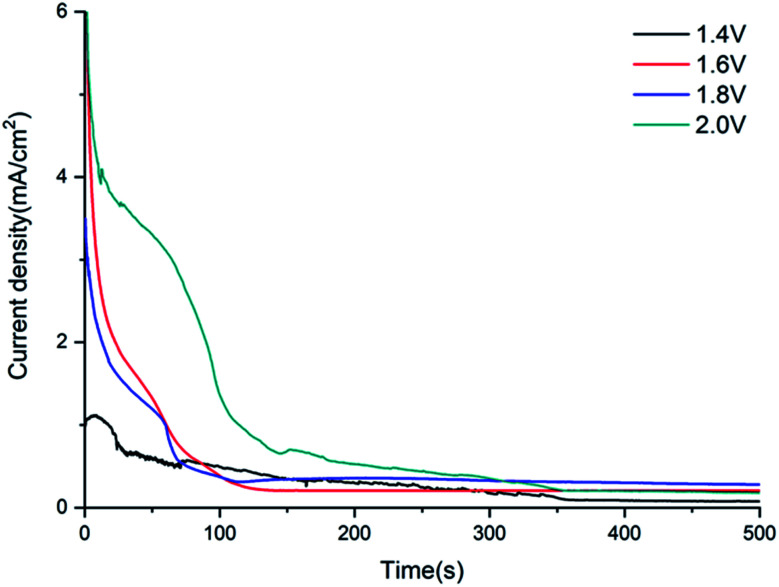
Current–time curves of GR electrodes at different potentials.

Fig. 3 illustrates the current–time curves traced during the potentiostatic treatment of GR electrodes at different constant potentials. It is shown that there is a little oxidation current with GR nanosheets on the surface of the ITO-PET substrate at 1.4 V potentiostatic potential within the initial 36 s, and the peak of anode current was about 0.6 mA cm^−2^. That is, the GR film may be oxidated and doped with SO_4_^2−^ ions on the ITO-PET film from 1.4 V to 2.0 V. Moreover, the current density gradually increases along with the rise of constant potential; that is, the growth rate of carbon nanoballs electrochemically stripped at higher potential is much faster than that at lower potential. In particular, as the constant potential moved to a more positive potential of 1.6 V, the current density increased sharply to two times, and the peak of the anode current was about 1.2 mA cm^−2^ at 50 s. This phenomenon might be attributed to the greater number and the faster growth rate of carbon nanoballs taking place on the surface of GR nanosheets when the constant potential increased to 1.6 V. And as the constant potential reached 1.8 V, the current density of the GR electrode was similar to that of the potentiostatic treated at 1.6 V. It indicated that the growth process of carbon nanoballs at 1.8 V was the same as at 1.6 V. The growth process of carbon nanoballs on GR films always goes through two steps: the rapid growth period and the steady saturation period. During the initial stage, the current density decreases sharply, and the initial rapid growth time is shortened within 100 s or so. It indicates that the nucleation rate in the initial step is much more rapid. This decrease of anodic current depends on the stripping of carbon nanoballs from GR sheets during the growth process. Firstly, GR nanobuds were produced during the induction period on the edge of GR sheets, then graphene nanobuds grew from the edge of sheets, and soon the GR sheets were coated with many nanobuds. Secondly, the secondary nucleation of GR nanobuds occurs already on the GR sheets; the GR nanobuds grew bigger and bigger, swelled to nanoballs, and then stripped from the GR sheets. It indicated that the growth of carbon nanoballs would not be continually increasing but finally level off. After the induction period, the current density keeps steady for a long time, and the exfoliation process of carbon nanoballs enters the saturated growth stage.

While the constant potential increased to 2.0 V, the peak of the oxidation current reached about 3.3 mA cm^−2^ at 50 s or so, which was nearly three times the current at 1.8 V. The reason may be that the peroxidation of graphene sheets at a higher potential of 2.0 V leads to the formation of a large amount of graphene oxide fragments, and the oxidation current increased significantly compared with that at 1.8 V. Hence, the growth of graphene at 2.0 V was very different from that at 1.8 V. This point can also be deduced from the micromorphology of the GR film at 2.0 V by SEM (Fig. 2(e)).

### 3.3 Fourier-transform infrared spectral analysis

**Fig. 4:**
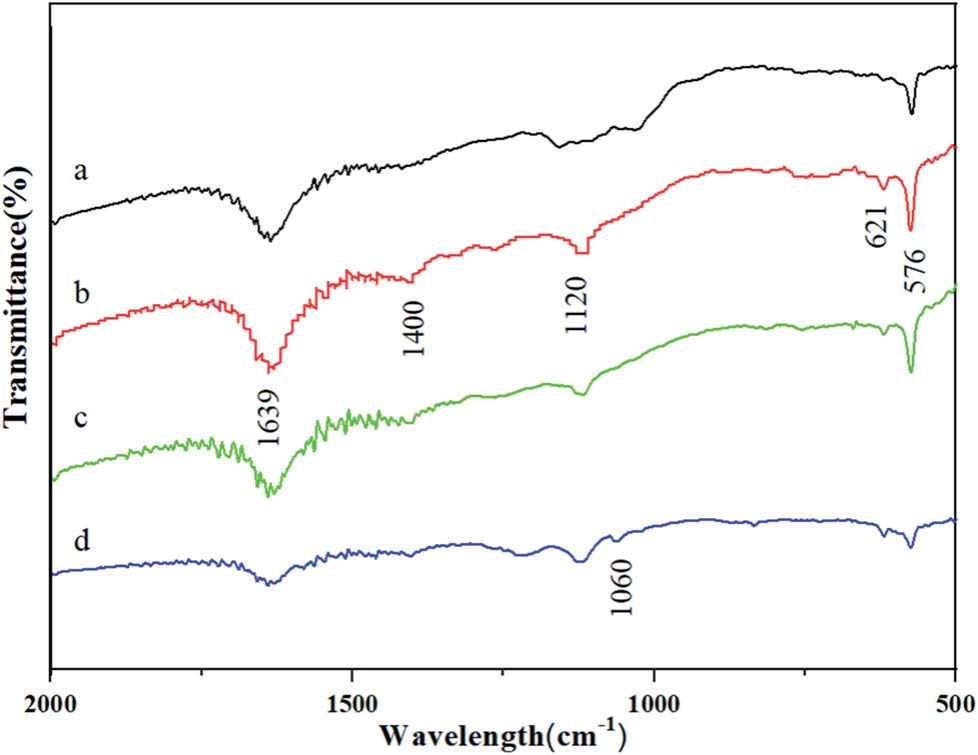
FTIR spectra of GR electrodes potentiostatic treated at (a) 1.4 V, (b) 1.6 V, (c) 1.8 V, and (d) 2.0 V.

The Fourier-transform infrared (FTIR) spectra of graphene electrodes at different constant potentials are shown in Fig. 4. The broad peak in the frequency range of 1639 cm^−1^ was attributed to the C=C stretching vibrations of the sp^2^ hybridized carbon bond, which was the same as the characterization peak of graphite. As the constant potential increases, the presence of carbon nanoballs like C_60_ in the GR sheets electrically treated at 1.6 V and 1.8 V is easily indicated in the FTIR spectrum with the appearance of two characteristic peaks at 1400 and 1120 cm^−1^ as presented in Fig. 4(b) and (c), which are associated with the tangential motion of carbon atoms of fullerenes. The rest two characteristic peaks at 621 and 576 cm^−1^ which are correlated with the radial motion of the carbon atoms of C_60_ and C_70_ were also detected. Then as the constant potential increased to 2.0 V, the graphene structure changed and new oxygen-containing groups were formed, which may be caused by the peroxidation of the graphene electrode at the higher potential. As shown in Fig. 4(d), the new absorption peak at 1060 cm^−1^ was corresponding to the C–O–C stretching deformation of graphene oxide. The reason is the peroxidation phenomenon of the graphene structure caused by higher potential.

### 3.4 X-ray diffraction analysis

**Fig. 5:**
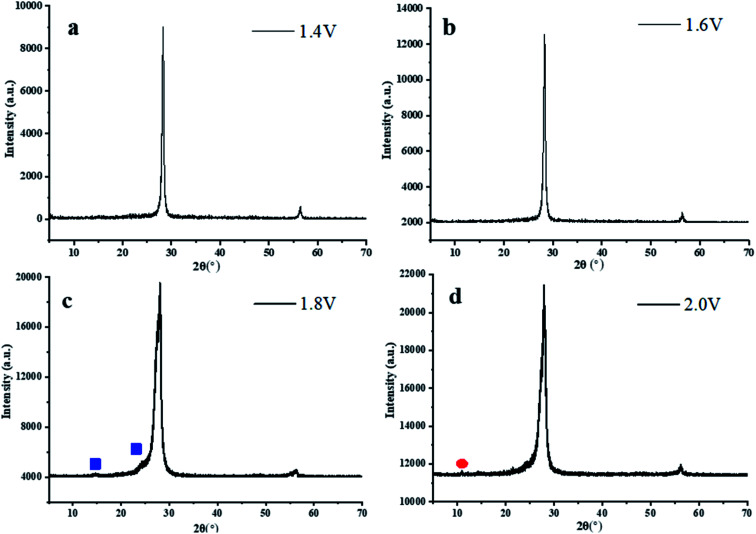
XRD of GR electrodes potentiostatic treated at (a) 1.4 V, (b) 1.6 V, (c) 1.8 V, and (d) 2.0 V.

The X-ray diffraction (XRD) patterns of the GR electrode at different potentials are shown in Fig. 5. A distinct diffraction peak at 28.3°, 56.5° was observed for the GR nanosheets under the potentiostatic treatment; the corresponding crystalline planes were (002), (004), which were similar to the crystallization peak of graphite at 26.5° (*d* = 0.336 nm), and 54.6°. Hence, the diffraction peaks of GR under an electric field significantly shift to a bigger angle, the crystal distance of GR sheets decreases due to the formation of smaller size graphene. And then, as the constant potential rose, the additional weak peaks at 14.6° and 24.3° were present in the pattern of GR at 1.8 V, and the corresponding crystalline planes were (002), (110), indicating the formation of many carbon nanoballs like C_60_ due to the incomplete oxidation/intercalation of graphene when the GR electrode is at a relatively higher potential. Meanwhile, a broad peak at 25.1–29.8° was found for the GR samples subjected to potentiostatic treatment of 1.8 V. It can be inferred that graphene nanosheets peeled off with the increase of constant potential. The degree of disorder of the original lamellar crystal structure increased under the effect of an electric field. When the constant potential was more than 1.8 V, the diffraction peak at 28° was obviously widened, and the degree of disorder further improved. Another detectable characteristic peak was observed in the patterns of the GR sample at 2.0 V, corresponding to (001) crystalline planes of graphene oxide (GO), which was located at approximately 10.8°, revealing a few graphene oxides achieved. During the oxidation process of graphene, a strong electric charge intercalated into graphene layers and lots of oxygen-containing groups were formed on the surface of graphene flakes. After the peroxidation of graphene under potentiostatic treatment of 2.0 V, large quantities of graphene fragments fall off, forming GO.

### 3.5 Raman spectroscopy analysis

**Fig. 6:**
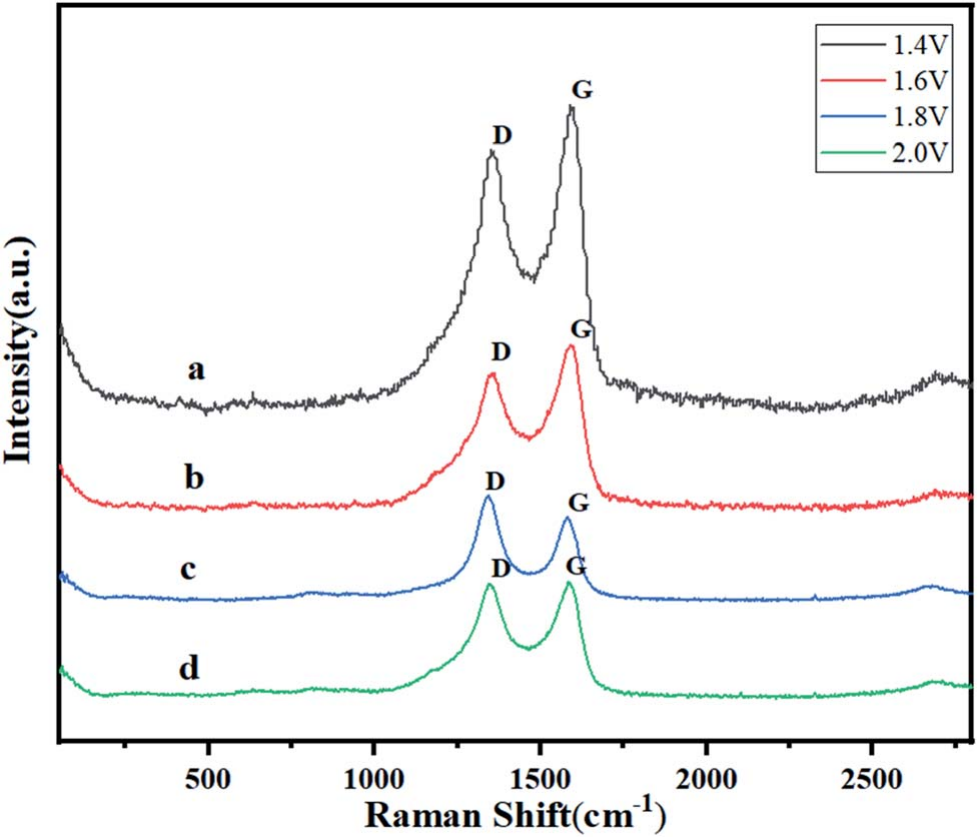
Raman spectra of GR electrodes potentiostatic treated at (a) 1.4 V, (b) 1.6 V, (c) 1.8 V, and (d) 2.0 V.

Raman spectroscopy, the inelastic scattering of matter to light, has played a central role in the scientific development of graphene and is the most important characterization tool for analyzing graphene-based materials to verify the success of covalent functionalization and to determine the quality of exfoliated graphene.^24^ It is a non-destructive technique that reveals the interactions between individual graphene sheets and functional groups. The Raman spectra of graphene electrodes at different constant potentials are shown in Fig. 6. The measured excitation wavelength was recorded at 532 nm and the laser power was 5 mW. As can be seen from the figure, all the curves show two intense peaks near 1350 cm^−1^ attached and 1580 cm^−1^, which are the two main characteristic peaks of the Raman spectra of graphene, the D peak and the G peak. In graphene, the G-peak originates from the first-order Raman scattering process of the double simplex plane transverse optical (TO)/longitudinal optical (LO) phonons in the center of the Brillouin zone, and the two sub-peaks in the Stokes and anti-Stokes Raman components of the D-peak are caused by an inelastic scattering event of the emitted photons in the double resonance Raman process or an elastic scattering event of the crystal defects.^25^ Where the D peak is activated by the edge of a nanosheet with structural defects comparable to or smaller than the laser spot is characteristic of graphene sp^2^ materials, and the G band is associated with defects in graphene. It is known that *I*(D)/*I*(G) is related to the defect density, but not linearly with the defect density, and it varies with the excitation energy. After calculation, the value of *I*(D)/*I*(G) increases gradually with the increase of constant potential, and then decreases slightly when the constant potential continues to rise to 2.0 V. It can be seen that the value of *I*(D)/*I*(G) is maximum when the constant potential is 1.8 V, *i.e.*, the more obvious the defect of the graphene sheet is at this time. We guess that the reason for the formation of this defect is that carbon nano-budding grows on the graphene sheet under the treatment of constant potential, and as the constant potential rises, the carbon nano-budding grows into nano-spheres, which causes the defect to become more and more obvious. As seen from the curves in Fig. 6(d), the intensity of the D peak is similar to that of the G peak, which is due to the oxidation of graphene at a higher constant potential of 2.0 V, resulting in the formation of a large number of GO fragments.

## 4. Conclusions

In this research, carbon nanobuds can be efficiently generated from graphene under a potentiostatic electric field. The results of this study showed that amounts of nanobuds were *in situ* growing on the surface of GR sheets as a constant potential of 1.4 V was added to the GR electrode. With the constant potential increasing, the nanobuds grew into the nanoballs, exfoliating from the surface of graphene sheets, whereas the peroxidation of graphene sheets occurred at a higher potential of 2.0 V, leading to the formation of a large amount of graphene oxide fragments. Therefore, the optimal processing parameter of the formation of carbon nanoballs was under the constant potential of 1.8 V for 500 s.

## Author contributions

Qi Qin and Jing Chen performed experiments and data analysis, Changze Wu and Yixue Wang developed the SEM technique, Fengyi Cao performed the XRD technique, Yunan Li and Zhongzhu Liu performed FTIR experiments, and Meng Song designed the experiments and coordinated the analysis.

## Conflicts of interest

The authors declare the following financial interests/personal relationships which may be considered as potential competing interests. Qi Qin reports that financial support was provided by Zhongyuan University of Technology. Qi Qin reports a relationship with Zhongyuan University of Technology that includes employment.

## Supplementary Material
